# Transport and retention of differently coated CeO_2_ nanoparticles in saturated sediment columns under laboratory and near-natural conditions

**DOI:** 10.1007/s11356-019-04965-x

**Published:** 2019-04-08

**Authors:** Laura Degenkolb, Urs Dippon, Silke Pabst, Sondra Klitzke

**Affiliations:** 1 Section Drinking Water Treatment and Resource Protection, German Environment Agency, Schichauweg 58, 12307 Berlin, Germany; 2 Department of Soil Science, Institute of Ecology, Berlin University of Technology, Ernst-Reuter Platz 1, 10587 Berlin, Germany

**Keywords:** Cerium dioxide mobility, Nanomaterial transport, Sediment column, Riverbank filtration, Colloidal stability, Hydrochemical conditions, Surface water

## Abstract

Where surface-functionalized engineered nanoparticles (NP) occur in drinking water catchments, understanding their transport within and between environmental compartments such as surface water and groundwater is crucial for risk assessment of drinking water resources. The transport of NP is mainly controlled by (i) their surface properties, (ii) water chemistry, and (iii) surface properties of the stationary phase. Therefore, functionalization of NP surfaces by organic coatings may change their fate in the environment. In laboratory columns, we compared the mobility of CeO_2_ NP coated by the synthetic polymer polyacrylic acid (PAA) with CeO_2_ NP coated by natural organic matter (NOM) and humic acid (HA), respectively. The effect of ionic strength on transport in sand columns was investigated using deionized (DI) water and natural surface water with 2.2 mM Ca^2+^ (soft) and 4.5 mM Ca^2+^ (hard), respectively. Furthermore, the relevance of these findings was validated in a near-natural bank filtration experiment using HA-CeO_2_ NP. PAA-CeO_2_ NP were mobile under all tested water conditions, showing a breakthrough of 60% irrespective of the Ca^2+^ concentration. In contrast, NOM-CeO_2_ NP showed a lower mobility with a breakthrough of 27% in DI and < 10% in soft surface water. In hard surface water, NOM-CeO_2_ NP were completely retained in the first 2 cm of the column. The transport of HA-CeO_2_ NP in laboratory columns in soft surface water was lower compared to NOM-CeO_2_ NP with a strong accumulation of CeO_2_ NP in the first few centimeters of the column. Natural coatings were generally less stabilizing and more susceptible to increasing Ca^2+^ concentrations than the synthetic coating. The outdoor column experiment confirmed the low mobility of HA-CeO_2_ NP under more complex environmental conditions. From our experiments, we conclude that the synthetic polymer is more efficient in facilitating NP transport than natural coatings and hence, CeO_2_ NP mobility may vary significantly depending on the surface coating.

**Electronic supplementary material:**

The online version of this article (10.1007/s11356-019-04965-x) contains supplementary material, which is available to authorized users.

## Introduction

Cerium dioxide nanoparticles (CeO_2_ NP) are used in various industrial applications, e.g., in diesel fuels, catalytic converters, and polishing agents (Garcia et al. 2005; Reed et al. 2014). As a result, increasing concentrations of these engineered NPs in the environment can be expected. In surface waters, CeO_2_ NP are predicted to reach concentrations in the range of 10^−2^ μg L^−1^ (Gottschalk et al. 2013). The presence of CeO_2_ NP in the environment was repeatedly shown to cause negative effects on organisms such as ammonia-oxidizing bacteria (García et al. 2012) or earthworms (Collin et al. 2014). Damage of human lung cells as well as oxidative stress can also be caused by CeO_2_ NP (Lin et al. 2006). An exposure to humans, for example via drinking water, should therefore be avoided. As riverbank filtrates are an important source for drinking water (Sprenger et al. 2017), it must be ensured that CeO_2_ NP entering the environment will be retained in these systems by natural filtration mechanisms, e.g., straining, adsorption, and attachment to biofilms. To understand the likelihood for transport of NP into environmental compartments such as groundwater, studies on the stability and the transport of NP in natural systems are important.

Many studies are currently undertaken on this topic elucidating the main factors governing NP transport in porous systems. Those factors include intrinsic NP properties (e.g., shape and size, Tiraferri and Borkovec 2015), hydrochemical properties of the mobile phase (e.g., pH and ionic strength, Praetorius et al. 2014) as well as characteristics of the pore system itself (e.g., pore size and connectivity, Cornelis et al. 2013; Fang et al. 2016; Lv et al. 2016; Troester et al. 2016). Additionally, organic molecules adsorbed to NP surfaces are of high importance for their colloidal stability: while bare CeO_2_ NP do commonly aggregate in most surface waters, the coverage of their surface with either synthetic or natural organic coating agents can stabilize CeO_2_ NP to different degrees (Booth et al. 2015; Dippon et al. 2018; Sehgal et al. 2005). A very effective manufactured stabilization agent is polyacrylic acid (PAA) as it exhibits a high negative surface charge stabilizing NP by electrostatic repulsion (Sehgal et al. 2005). Authors showed that PAA-coated CeO_2_ NP were stable even at Ca^2+^ concentrations up to 6 mM, which could potentially result in high transport of NP in column experiments (Chanteau et al. 2009; Petosa et al. 2013). In contrast, natural coating agents such as humic acid (HA) and fulvic acid (FA) seem to stabilize CeO_2_ NP in a broad pH range but the presence of divalent cations such as Ca^2+^ can cause bridging effects and strong aggregation of NP (Oriekhova and Stoll 2016; Stankus et al. 2011).

Nanoparticles released in the environment will most likely be coated with natural organic matter (NOM); synthetic coatings might be replaced or covered by NOM (Louie et al. 2016) which may change NP stability (Davis and Gloor 1981; Tipping and Higgins 1982). In comparison to uniform, synthetic coatings used to stabilize NP suspension in industrial processes, natural organic matter consists of a variety of macromolecules that form a heterogeneous coating on NP surfaces. This will most likely lead to different stability and therefore mobility of naturally and synthetically coated NP. However, the different influence of synthetic compared to natural occurring polymers on CeO_2_ NP transport has not been studied in detail so far. Laboratory column experiments investigating the influence of one or more of the before-mentioned factors showed that a transport of NP through sediments or soils takes place when NP are colloidally stable, but NP will be retained in systems with high ionic strength (e.g., Liang et al. 2013; Petosa et al. 2013). Organic matter concentrations and ionic strength affect NP attachment to sediment surfaces: increasing ionic strength enhances the attachment, while the presence of organic matter reduces it (Geitner et al. 2017). Therefore, we tested the influence of waters with different ionic strength on NP transport comparing different organic matter coatings on CeO_2_ NP. We hypothesize that polyacrylic acid-coated CeO_2_ NP will be more stable in natural surface water as they are less susceptible to enhanced Ca^2+^ concentrations than natural organic matter-coated CeO_2_ NP. Thus, a higher transport of PAA-CeO_2_ NP is expected to occur in saturated sediment columns.

In addition, processes determining the transport behavior of NP can vary significantly from lab studies to environmental systems. As an example, Markus et al. (2015) stated that the aggregation processes taking place differ in lab experiments compared to environmentally relevant systems. The importance of heteroaggregation on NP fate in natural surface water of a mesocosm experiment has been shown by Espinasse et al. (2018). Furthermore, biological activity in environmental systems may lead to more versatile attachment sites than present in well-defined laboratory systems. We expect that this increased complexity will cause a stronger retention in natural systems compared to lab studies and should therefore be taken into account when assessing a potential risk of NP transport.

In the first part of our study, we compared the mobility of PAA-coated CeO_2_ NP (PAA-CeO_2_ NP) with CeO_2_ NP coated by NOM or HA (NOM-CeO_2_ NP and HA-CeO_2_ NP) in laboratory sand columns under saturated conditions. The experiments were conducted in deionized water as well as natural surface water with different Ca^2+^ concentrations to account for different types of water. In the second part, the transport of HA-CeO_2_ NP was investigated in a more complex bank filtration system in semi-technical scale and these results were compared to the results of laboratory column experiments. With this approach, our study aimed at identifying differences in the transport of synthetically and naturally coated CeO_2_ NP in sandy sediments in the presence of different surface waters. The relevance of findings from laboratory studies was validated by an outdoor bank filtration experiment.

## Material and methods

### Nanoparticles and coatings

Polyacrylic acid-coated CeO_2_ NP (Partikular GmbH, Germany) with a hydrodynamic diameter (HDD) of 74 nm were produced by laser ablation from bulk material directly within PAA solution (Sigma Aldrich, *M*_*w*_ = 2100). Therefore, particle surface modifications due to intermediate synthesis steps or residues from synthesis chemicals can be excluded.

Pristine CeO_2_ NP for coating with NOM and HA were purchased from Nyacol Inc. (USA). The uncoated particles with an HDD of 80 nm were stabilized in acetic acid at pH 2. These particles were coated prior to experiments with either NOM or HA using the following procedures: (a) Nordic reservoir-NOM was purchased from IHSS (International Humic Substance Society, USA, Nordic reservoir organic matter, batch 1R108N) as powder with 1 g NOM containing 0.31 g *C*_org_. For stock solutions, 0.02 g NOM powder was dissolved in 100 mL ultrapure water. Pristine CeO_2_ NP were coated by adding 10 mL of a 500 mg L^−1^ CeO_2_ NP suspension into 90 mL of 200 mg L^−1^ NOM solution and subsequent stirring for 48 h at 4 °C in the dark. Finally, the pH of the suspension was adjusted to pH 7 using dilute KOH (analytical grade, Merck, Germany). (b) HA sodium salt (Sigma Aldrich) solutions were prepared like the NOM solution but filtered (0.45 μm, regenerated cellulose) before addition of the NP. For the bank filtration experiment, HA was prepared in shares of 4 g per 4.8 L water and centrifuged before filtration (Schleicher and Schuell filter paper).

Prior to the experiments, the differently coated NP suspensions were diluted in the target water to 4 mg L^−1^ of CeO_2_ leading to different particle properties depending on hydrochemical conditions (Table 1). All solutions containing NOM or HA were stored at 4 °C in the dark and used within 1 week.

**Table 1:** Nanoparticle properties (hydrodynamic diameter (HDD) before and after the experiment and ζ-potential values; mean and standard deviation from three replicates) and water conditions (electrical conductivity (EC) and pH) of the laboratory column experiments

Medium	DI water		Soft surface water			Hard surface water	
Coating	PAA	NOM	PAA	NOM	HA	PAA	NOM
HDD (Z-Ave) before (nm)	74	79	76	64	105	87	590
SD	1	1	0	1	5	2	37
HDD (Z-Ave) after (nm)	70	78	73	88	156	87	> 3000^a^
SD	1	0	1	1	1	2	699
HDD (Vol-Pk 1) before (nm)	55	55	57	45	86	54	70
SD	3	6	2	5	14	15	22
HDD (Vol-Pk 1) after (nm)	56	49	52	64	75	53	–^b^
SD	2	6	2	2	30	3	–^b^
ζ-potential (mV)	− 48.1	− 34.8	− 22.2	− 21.6	− 18.0	− 18.7	− 17.9
SD	3.0	12.0	0.8	2.1	0.9	1.0	1.1
EC (μS cm^−1^)	0.31	0.31	527	786	505	995	1001
pH	7.5	6.5	7.6	7.9	6.9	7.8	7.9

^a^PdI > 0.7, therefore DLS data not reliable

^b^Not evaluated due to particle sedimentation

### Properties of the water-saturated sediment system

The transport of CeO_2_ NP was investigated under water-saturated conditions in quartz sand columns. As mobile phase, we used natural pond water from a sand filtration pond located at the property of the Federal Environment Agency, Berlin. For laboratory column experiments, the water was sampled from the pond and filtered (< 0.45 μm, Nylon, Whatman, GE Healthcare) prior to each experiment and the main ion concentrations were measured and found to be constant during all sampling time points (Online Resources, Fig. S1). To investigate the influence of the water hardness on NP transport, we used differently hard water: deionized (DI) water (Ca^2+^ concentration 0 mM), soft surface water (DI water-diluted pond water with a Ca^2+^ concentration of 2.2 mM), and hard surface water (pond water with a Ca^2+^ concentration of 4.5 mM). In the context of this study, the terms “soft” and “hard” were chosen to differentiate between the two Ca^2+^ concentrations and do not correspond to any classification system. Columns of the outdoor experiments were located in the sand filtration pond and flushed with surface water throughout the year. For our study, we diluted the surface water to soft water conditions (Ca^2+^ concentration 2.2 mM) using DI water.

All columns were filled with medium grain-sized sand (*d*_50_ = 1.8 mm; grain size distribution Fig. S2 in Online Resources). For lab experiments, the sand was washed with DI water and then filled into columns under water-saturated conditions to avoid trapping of air bubbles. Subsequently, the columns were equilibrated with particle-free water for 48 h prior to particle injection to ensure constant boundary conditions for all sand columns. The outdoor columns were filled with sediment 3 months prior to the start of the experiment and then flushed with natural, undiluted pond water. Therefore, a well-established ecological community can be expected in the outdoor columns, which was confirmed by microscopy images showing a high number of diatoms (Degenkolb et al. 2018). Two days prior to the NP transport experiments, the flushing water was changed to soft surface water to adjust the desired hydrochemical conditions.

### Laboratory column experiments

The laboratory columns were made of glass, equipped with polytetrafluoroethylene (PTFE) end pieces and had a length of 18 cm and a diameter of 4.3 cm. They were operated in upward flow to reduce the possibility of air entrapments. The flow rate was set to 0.5 mL min^−1^, equivalent to a pore water velocity (Darcy velocity) of 1.3 m day^−1^ using peristaltic pumps. A tracer experiment with NaCl was done prior to each NP transport experiment measuring the electric conductivity (TetraCon, WTW, Germany) in the column outflow. From this, an effective porosity (*η*_eff_) between 0.35 and 0.38 was calculated which was used to normalize the sampling time to the unit of “pore volumes” (PV).

The NP transport experiments were conducted in duplicates at room temperature. A CeO_2_ NP suspension with a final Ce concentration of 4.0 ± 0.6 mg L^−1^ was added to the desired water (DI, soft or hard surface water) one hour before start of the experiment. After this time, the suspension was injected at a constant flow velocity (0.5 mL min^−1^) for 3 PV. It should be noted that the pH of the added particle suspensions slightly differed between the experiments (Table 1). This was mainly caused by the different pH values of the NOM-, PAA- and HA-CeO_2_ NP stock suspensions and the buffering capacity of the applied water. After NP addition, flushing was done with the respective NP-free water for 13 PV. The outflow of the columns was sampled in intervals of 40 min in glass tubes. Aliquots of the water samples were used for cerium and total organic carbon (TOC) quantification (vario TOC cube, Elementar, Germany). As the TOC concentration showed the same transport behavior as the NaCl tracer (i.e., similar retention under identical flow velocity), this value can be interpreted as a tracer measured parallel to the NP injection indicating whether NP transport was retained or not. Upon termination of the experiment, the sand from the columns was sampled in 1 cm sections and analyzed for cerium content.

### Outdoor column experiments

To assess the transferability of results about CeO_2_ NP transport from laboratory columns to more complex environmental conditions, the facility for the simulation of riverbank and slow sand filtration (SIMULAF) located at the German Environment Agency in Berlin was used. This facility is described in more detail in Degenkolb et al. (2018). In brief, we used two water-saturated columns filled with the same medium grain-sized sand as used in laboratory column experiments. For experiments with NP, we diluted the water to an electric conductivity (EC) of 250–520 μS cm^−1^ by adding DI water equivalent to soft surface water conditions in the lab. Fluctuations in the conductivity were caused by heavy rain events. Physicochemical parameters of the diluted pond water are summarized in Table 2. In contrast to the lab experiments, the pond water was not filtered and therefore, the presence of natural colloids larger than 0.45 μm can be expected which is demonstrated by larger Fe concentrations in the mobile phase of outdoor columns (Table 2). The columns with a length of 1.5 m and a surface area of 1 m^2^ had sampling ports in 15, 30, 50, and 90 cm depth as well as at the outflow (Online Resources, Fig. S3). In 15, 30, 50, and 90 cm depth, water was sampled continuously with a pump rate of 0.5 L h^−1^. Samples were taken with an autosampler every 30 min for the first 24 h. Afterwards, the sampling interval was increased stepwise to 60 min (day 2), 90 min (days 3–4), and 6 h (days 5–30). The samples were collected in glass vessels and subsequently stored in plastic tubes at 10 °C until analysis. Additionally, samples from the outflow were taken manually once or twice a day directly into plastic tubes as the possibility for breakthrough in this depth was lowest. Electric conductivity and temperature were monitored over the whole experimental period in the column supernatant and EC also in the outflow (Online Resources, Fig. S4).

**Table 2:** Composition of water used in laboratory column experiments. In the outdoor column experiment, the water composition roughly matched the soft surface water, but fluctuations in concentrations were caused by changing weather conditions. Ion concentrations were measured by ion chromatography (Metrohm, Germany)

Parameter	DI water	Soft surface water/Outdoor columns^a^	Hard surface water
Electrical conductivity (μS cm^−1^)	0.055	520/250–520	850–950
pH	–^b^	7.7	7.7
Al (mM)	bdl	0.00	0.00
Ca (mM)	bdl	2.2	4.5
Fe (mM)	bdl	0.00/0.11	0.00
K (mM)	bdl	0.06	0.1
Mg (mM)	bdl	0.34	0.7
Na (mM)	bdl	1.25	2.5
Cl (mM)	bdl	1.19	2.4
SO_4_ (mM)	bdl	1.30	2.6
NO_3_ + NO_2_ (mg L^−1^)	bdl	0.03	0.05
NH_4_ (mg L^−1^)	bdl	0.01	0.01

*bdl* below detection limit

^a^Average values for dry weather conditions. Where lab and outdoor column experiments differed from each other noteworthy, two numbers are given (lab/outdoor)

^b^Not measurable due to low conductivity

The outdoor experiment was conducted from June 26 to July 25, 2017. A filter velocity of 0.5 m day^−1^ (i.e., Darcy velocity ≈ 1.45 m day^−1^) was kept constant over the entire experiment as well as during the tracer experiment. We used NaCl solution (EC in the supernatant = 2.5 mS cm^−1^) as a tracer and measured EC in all depths of the columns.

Prior to NP injection, samples of the water head of the sediment columns were collected as blank samples and to describe hydrochemical characteristics of the mobile phase. Cerium dioxide NP were injected into 100 L of diluted pond water in the column supernatant with a final Ce concentration of 4.3 mg L^−1^. After gentle stirring, three replicates were taken from each column supernatant. We waited until complete infiltration of the NP dispersion before adding diluted pond water as the mobile phase of the transport experiment. Sampling was conducted over a period of 30 days. During this time, two intense rainfall events significantly reduced the EC of the mobile phase (Online Resources, Fig. S4) but did not affect the flow velocity due to the constant pumping. This phenomenon was expected to influence the colloidal stability and thus the transport behavior of CeO_2_ NP and was therefore included in the evaluation of the experiment.

After termination of the experiment, sand samples were taken from 0 to 30 cm depth in 5 cm intervals and from 40 to 90 cm depth in 10 cm intervals measured from the sand surface. Cerium dioxide NP were extracted and quantified as described below.

### Analytical techniques

#### Particle size and ζ-potential

The particle size and ζ-potential of CeO_2_ NP were measured by dynamic light scattering (DLS) with a Zetasizer Nano ZS (Malvern Instruments, UK) and ζ-potentials were calculated based on the electrophoretic mobility of the NP (using the Helmholtz-Smoluchowsky equation). The NP size in different media was investigated in batch experiments prior to and at the end of column experiments (Table 1). All NP sizes indicated in this study are intensity-weighted least square sum *Z*-average values with PdI ≤ 0.3, which corresponds to a broad monodisperse distribution. These values are suitable to observe changes in NP size (e.g., caused by aggregation). However, as they are biased to larger particle sizes, the volume-weighted largest peak is also given in Table 1.

#### Digestion of Ce in aqueous samples

Aqueous suspensions taken from the column outflow of laboratory experiments and different depths of the outdoor columns, respectively, were sampled in 10 mL aliquots and dried at 40 °C for 12 h. The residues were then digested in the microwave using a modified aqua-regia digestion with HNO_3_ and HCl in a ratio of 3:1 (*v*/*v*). In detail, 6 mL of 65% HNO_3_ and 2 mL of 32% HCl were added to the dried sample and digested for 20 min at 160 °C in the microwave (Mars 5x, CEM GmbH) in PTFE or tetrafluorethylene-perfluorether copolymer (PFA) pressure vessels. Digested samples were filtered (Whatman filter paper) to remove possible quartz particles.

#### Digestion of Ce in sediment samples

Sediment samples of the columns were analyzed for Ce content to measure the amount of retained CeO_2_ NP. Acid digestion of the complete sediments yielded high background Ce concentrations with a large standard deviation, exceeding the cerium concentration applied in the form of NP (Online Resources, Fig. S5). Therefore, we developed a selective extraction technique based on established methods for the extraction of exchangeable ions (Filgueiras et al. 2002). An amount of 5 g wet sand was weighted into vials and a volume of 12 mL ultrapure water was added. These samples were sonicated for 60 min in an ultrasonic bath, and subsequently, 10 mL of the supernatant were pipetted for digestion as described for aqueous suspensions. The recovery of cerium by this method was 80% (Online Resources, Fig. S5).

#### Cerium quantification

All digested samples were analyzed for Ce concentrations by inductively coupled plasma optical emission spectroscopy (ICP-OES, PerkinElmer Optima 8300) in duplicates. Quantification limits, determined according to DIN 32645, were 2 μg L^−1^ in the measurement solution using the wavelengths 413.764 nm, 418.660 nm, and 401.239 nm. The measurement procedure followed the recommendation of ISO 11885 standard using 5% HNO_3_ in the matrix. As lab internal standard, ytterbium was used.

For all experiments, a mass balance was calculated from the mass of cerium broken through the column and the mass retained in the sediment (Online Resources, Eq. 1). The mass in aqueous samples of lab experiments was calculated by multiplying the sampling volume with the measured concentrations. As we collected the complete outflow and measured every second sample, we multiplied the mass of each sampling point by 2, assuming equal concentrations in neighboring samples. For calculation of the mass in sediments, the effective porosity of *η*_eff_ = 0.35 was used for all calculations and the measured values were corrected by the recovery rate of the digestion method.

## Results and discussion

### Nanoparticle properties

Polyacrylic acid-coated CeO_2_ NP had an HDD of < 90 nm in all tested water conditions (i.e., deionized, soft surface, hard surface water) and were stable over the course of the experiments (Table 1). In contrast, NOM-CeO_2_ NP had a stable HDD of < 80 nm in DI and soft surface water but aggregated in hard surface water (> 500 nm). In DI water, PAA-CeO_2_ NP had a more negative ζ-potential than NOM-CeO_2_ NP (− 48 compared to − 35 mV). In soft and hard surface water, ζ-potentials of NOM- and PAA- CeO_2_ NP were comparable (− 22.2 and − 21.6 mV in soft surface water and − 18.7 and − 17.9 mV in hard surface water, respectively, Table 1). For PAA-CeO_2_ NP, this decrease in ζ-potential did not change NP size. Polyacrylic acid is a well-known and effective stabilizing coating agent maintaining particle stability in Ca^2+^ concentrations up to 10 mM (Chanteau et al. 2009) which was confirmed by our measurements. However, the decreasing ζ-potential with increasing ionic strength suggests that not only electrostatic but also steric stabilization mechanisms might be responsible for the high stability of PAA-CeO_2_ NP. In contrast to PAA-CeO_2_ NP, NOM-CeO_2_ NP size increased in hard surface water. Probably, the bridging effect of Ca^2+^ between NOM-coated NP surfaces increased the aggregation process (Philippe and Schaumann 2014). As ζ-potentials seem to be not the determining parameter for NP stabilization, we conclude that different degrees of Ca^2+^ bridging were responsible for the different stability of synthetically and naturally coated NP. The stabilization of CeO_2_ NP in our study by PAA may be supported by high amounts of access PAA in NP suspension as described below (“Transport in deionized water”).

The surface properties of HA-CeO_2_ NP in soft surface water, which were injected into laboratory as well as the artificial bank filtration columns, were measured in the beginning and at the end of the lab column experiment. The HDD of HA-CeO_2_ NP slightly increased during column experiments from 105 to 156 nm and the ζ-potential (− 18 mV) was less negative compared to PAA- and NOM-CeO_2_ NP (Table 1). Due to the presence of natural inorganic and organic colloids in the pond water of outdoor columns, DLS measurements were not applicable in the large-scale experiments.

### Transport of differently coated CeO_2_ NP in laboratory column experiments

**Fig. 1:**
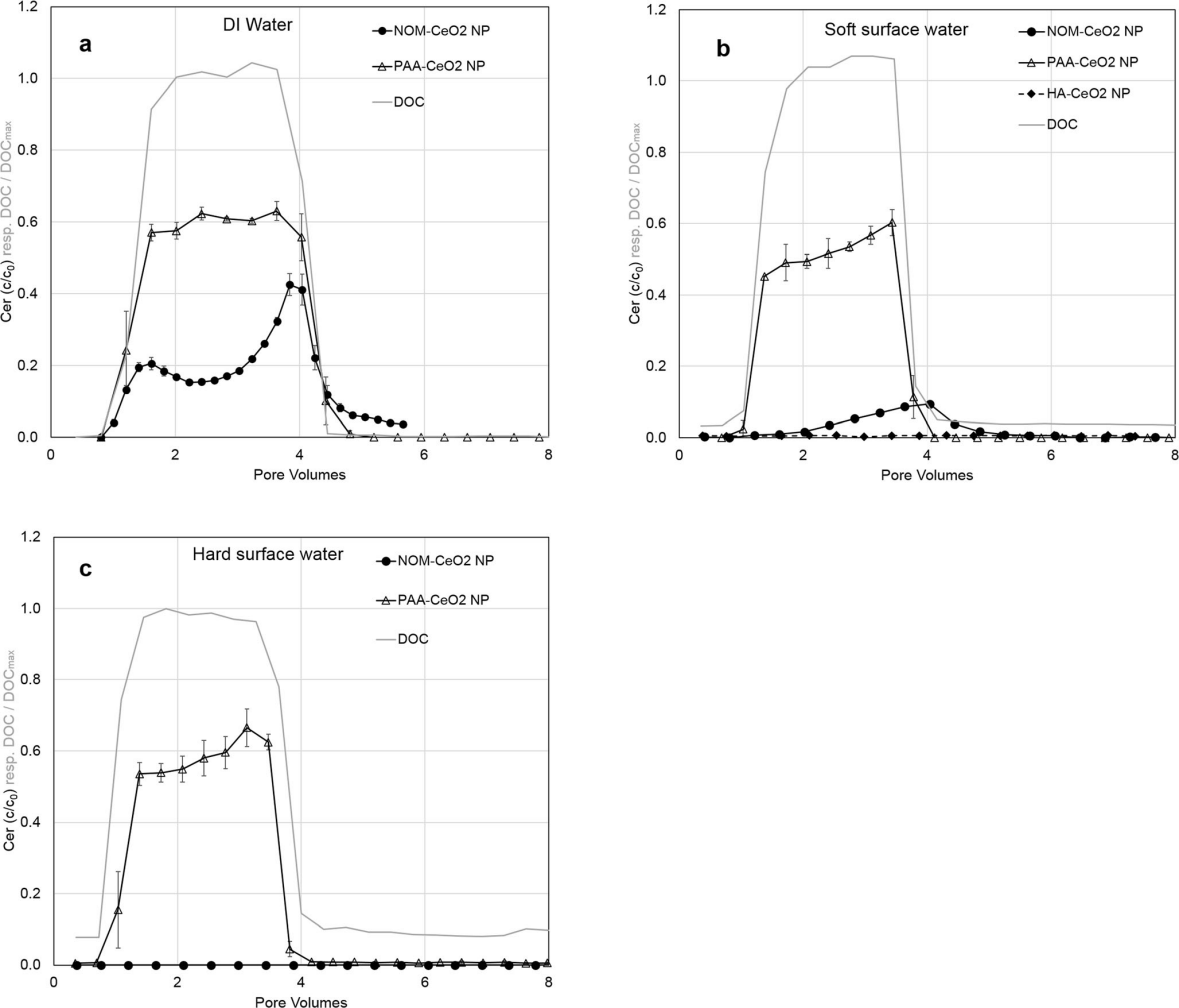
Normalized cerium concentrations in the outflow of the laboratory columns as a function of pore volumes. Circles represent NOM-CeO_2_ NP, triangles PAA-CeO_2_ NP and diamonds HA-CeO_2_ NP; the mobile phase was DI water (**a**), soft surface water (**b**) and hard surface water (**c**). The grey line represents the TOC concentration which can be interpreted as conservative tracer in this context. Errorbars depict the range (maximum and minimum values) of duplicate columns

Different organic coatings as well as varying hydrochemical conditions played an important role in the transport of CeO_2_ NP in saturated sediment columns (Fig. 1, error bars depict observed maximum and minimum value). Synthetically coated PAA-CeO_2_ NP showed more breakthrough (50–60 %) and lower retention than NOM-coated CeO_2_ NP (0–27 %; Online Resources, Table S1). Furthermore, the influence of Ca^2+^ was generally more pronounced for NOM-CeO_2_ NP compared to PAA-CeO_2_ NP.

#### Transport in deionized water

Breakthrough in DI water was observed for both PAA- and NOM-CeO_2_ NP (Fig. 1a). The strongly negative ζ-potentials and colloidal stability of the NP in this medium favored their transport. Nevertheless, both kinds of CeO_2_ NP were partly retained at the sediment-water-interface (SWI), with retention being stronger for NP coated with NOM. While NP coated with PAA showed a breakthrough of 60% of the initially injected Ce, only 27% of the NOM-CeO_2_ NP broke through (Online Resources, Table S1).

Polyacrylic acid-coated CeO_2_ NP showed almost no retardation in comparison to the tracer (TOC-concentration, Fig. 1a). This transport behavior is similar to the constant breakthrough of PAA-coated CeO_2_ NP observed by Petosa et al. (2013) in quartz sand columns. In contrast to this steady-state behavior, NOM-CeO_2_ NP showed a dynamic breakthrough increasing over the course of the experiment (Fig. 1a) which suggests the blocking of attachment sites with increasing NP injection (Bradford et al. 2011). Nevertheless, a decrease in NP concentration after approximately 1.5 PV was observed in our study for NOM-CeO_2_ NP in DI water. Decreasing breakthrough over time is commonly interpreted as a consequence of mechanical filtration processes leading to pore clogging (Petosa et al. 2013). However, aggregation of CeO_2_ NP in DI water was not observed (Table 1) suggesting that this explanation might not be valid for our experiment as particles are considerably smaller than the pore system. Therefore, surface attachment processes must play a dominating role for the reduced breakthrough and transport of CeO_2_ NP through our columns, comparable to observations in other studies with CeO_2_ NP (e.g., Geitner et al. 2017). The dynamic breakthrough profile of NOM-CeO_2_ NP is more similar to curves observed for systems with a higher variety of attachment sites as described in the study by Petosa et al. (2013). In our study, a higher variety of functional groups is expected for the NOM coating compared to the PAA coating as PAA has only carboxyl groups while Nordic reservoir-NOM consists of various molecules with different functional groups (Ritchie and Perdue 2003). This enhanced variability of NP led to more possibilities for particle-sediment interactions, hence increasing retardation processes, an effect that has already been described by Bolster et al. (1999). Furthermore, NOM-CeO_2_ NP showed a tailing after 5 PV which suggests that NOM-CeO_2_ NP were reversibly attached in a secondary energy minimum under low ionic strength conditions. In contrast to our results, Geitner et al. (2017) observed a generally lower steric stabilization effect for linear and more uniform coating molecules (such as PVP) compared to larger, more heterogenic structures (such as HA and NOM). This underlines that steric stabilization alone might not be the prevailing stabilizing mechanism for PAA coating, but electrostatic effects are also relevant.

Another factor that might have increased the transport of the PAA-CeO_2_ NP was the higher background TOC concentration in PAA-CeO_2_ compared to NOM-CeO_2_ NP suspension (115 ppm compared to 17 ppm) which might have enhanced the repulsion between sediment surfaces and NP and led to competition for sorption sites. In a study by Chen et al. (2012), the addition of humic acids (1–10 mg L^−1^) to sand columns led to increased TiO_2_ NP elution, which also suggests an influence of higher TOC concentrations in our study.

**Fig. 2:**
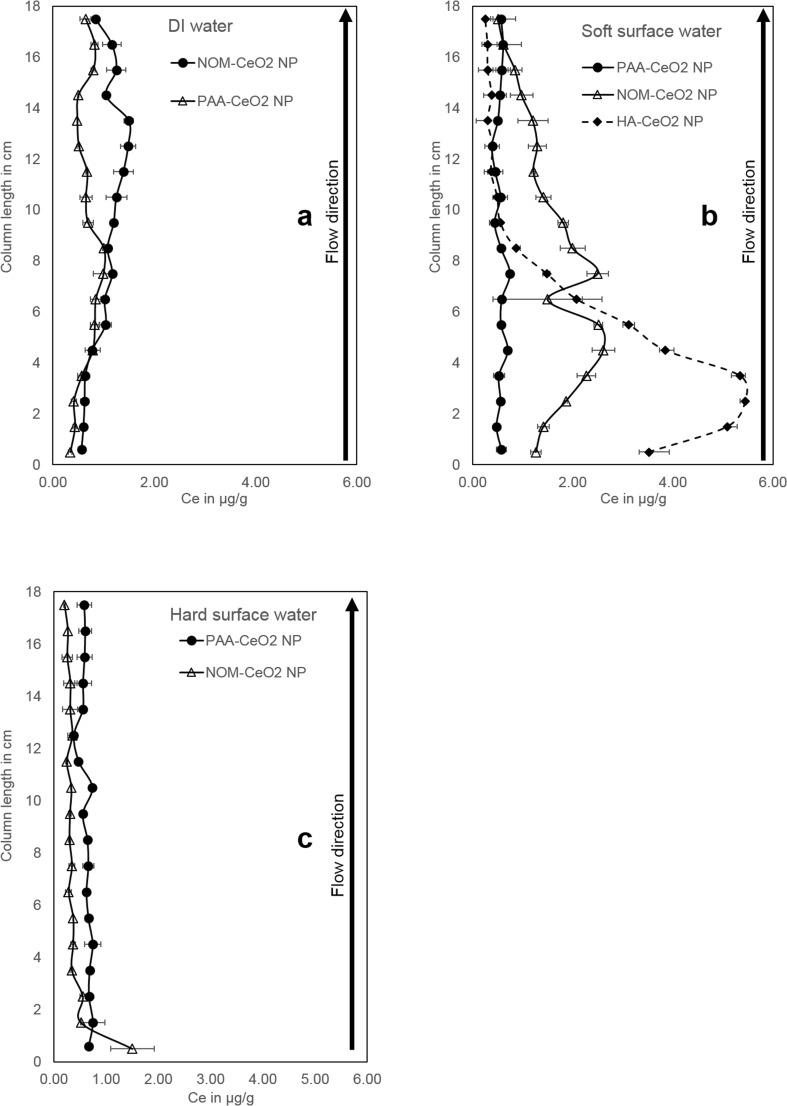
Cerium content in filter sand of the lab columns flushed with DI water (a), soft surface water (b), and hard surface water (c) for PAA- CeO2 NP (circles), NOM-CeO2 NP (triangles), and HA-CeO2 NP (diamonds). Errorbars depict the range (maximum and minimum values) of duplicate columns

The stronger retention of NOM-CeO_2_ NP was further demonstrated by the retention profiles (Fig. 2a): higher Ce contents in the sediments were measured in the case of NOM-coated NP compared to PAA-CeO_2_ NP. Our extraction method which included the separation of CeO_2_ NP from the sediment by ultrasonic bath treatment had a recovery of only 80%. Hence, the Ce contents cannot be interpreted as absolute concentrations, but a clear trend for the distribution of Ce in the columns is visible. Interestingly, NOM-CeO_2_ NP seem to accumulate in the upper part of the column close to the outflow. This is evidence of detachment processes, which are also indicated by the tailing of the breakthrough curve. Typically, particles attached in a shallow secondary energy minimum are detachable for example by hydrochemical changes and are then slowly transported through the column (Braun et al. 2015).

#### Soft surface water

In soft surface water (i.e., Ca^2+^ concentration of 2.2 mM), PAA-CeO_2_ NP broke through without any retardation (Fig. 1b) and with a recovery of 50% of initially injected NP (Online Resources, Table S1). The slight increase of breakthrough over time indicates saturation of attachment sites. In contrast, NOM-coated NP showed a total breakthrough of less than 10%, which appeared obviously retarded. The shift in breakthrough compared to the tracer (TOC concentration) can be explained by equilibrium sorption processes while the tailing of the breakthrough curve indicates non-equilibrium retardation processes typically occurring at the SWI (Kumahor et al. 2016). Enhanced retention, as shown by the low recovery in the effluent, might be a result of enhanced Ca^2+^ concentrations in soft surface water compared to DI water. Calcium is highly effective in screening the surface charge of NP as well as of sediment surfaces, and this reduces electrostatic repulsion, thus increasing the likelihood of deposition processes (Derjaguin and Landau 1941; Verwey and Overbeek 1948). Petosa et al. (2013) observed no effect of Ca^2+^ on PAA-CeO_2_ NP for concentrations below 6.7 mM which is in line with the unaffected transport behavior of PAA-CeO_2_ NP in our study. Instead, NOM-CeO_2_ NP were already affected by a Ca^2+^ concentration of 2.2 mM. This supports the hypothesis of a higher stability of the synthetic coating agent PAA compared to natural coatings. In the presence of monovalent cations, Li et al. (2013) found a complete breakthrough of NOM-coated CeO_2_ NP up to NaCl concentrations of 3 mM. This underlines the strong effect of divalent cations on NP stability as present in the water phase of our study.

While the retention of PAA-CeO_2_ NP was equally distributed over the whole length of the column, NOM-CeO_2_ NP were mainly retained between 3 and 8 cm depth (Fig. 2b). Compared to the experiments in DI water, NOM-coated CeO_2_ NP were transported over a shorter distance in soft surface water, due to their lower stability and stronger interaction with the sediment surfaces in the presence of higher ionic strength. This is in line with the low Ce concentrations in the outflow of the column. The NP accumulation at a distance from the column inlet demonstrates that non-equilibrium sorption and detachment of NP retained in a secondary energy minimum caused a slow NP transport through the column.

As both CeO_2_ NP showed a similar HDD of < 80 nm under soft surface water conditions before application to the column, retention is not expected to be caused by physical straining processes (i.e., deposition of NP aggregates in small pore spaces, e.g., in grain-grain-contact zones). Straining is dependent on the ratio of colloid to median grain size diameter (*d*_50_). Bradford et al. (2002) found that colloid transport was not influenced by straining in sand columns with a *d*_50_ as large as 0.71 mm and a colloid size smaller than 0.45 μm. As we have a larger *d*_50_ (i.e., 1.88 mm) and smaller initial particle sizes (i.e., 75 nm), straining processes are unlikely to occur for non-aggregated NP. Instead, attachment of CeO_2_ NP to the sediment surfaces supported by the bridging effect of Ca^2+^ were responsible for NP retention. Those effects have been observed for example by Wang et al. (2015) for the transport of PVP-coated Ag NP in soils. Bridging, however, seemed to play a minor role for PAA-CeO_2_ NP. Chanteau et al. (2009) explained the high stability of those NP with the formation of anionic adlayers on the NP surfaces leading to both electrostatic and steric stabilization (Sehgal et al. 2005). In contrast, Torkzaban et al. (2012) observed deposition of PAA-coated quantum dots on sand for Ca^2+^ concentrations as low as 1 mM. The authors explained this by bridging complexation of the carboxylic group of PAA forming bridges between NP and solid surfaces. They used lower particle concentrations (1.4 × 10^11^ NP L^−1^ compared to 6.1 × 10^13^ NP L^−1^ in our study) which might partially explain the different results. A recent study by Dippon et al. (2018) compared the colloidal stability of NOM- and PAA-coated CeO_2_ NP in batch experiments, finding a stronger stabilizing effect for NOM compared to PAA. Their experiments were conducted with lower PAA background concentrations (77 ppm compared to 115 ppm in our study). This would lead to the conclusion that the destabilization of PAA-CeO_2_ NP by Ca^2+^ might be dependent on the ratio between Ca^2+^ and PAA in the system. Probably, free PAA can remove Ca^2+^ from the solution by reactions with the carboxylic groups, thereby preventing a destabilization of CeO_2_ NP by Ca^2+^. Additionally, PAA as a manufactured polymer can have different chain lengths which might be crucial for the occurrence of steric stabilization processes (Ghosh et al. 2011). Therefore, it is difficult to compare the results of studies that used different PAA-coated NP when information about background TOC and chain length is not provided.

In soft surface water, we investigated the transport of CeO_2_ NP with a second kind of natural coating agent, namely humic acids (Fig. 1b). Due to the availability of large quantities of this coating agent, we chose it for the experiments in the large-scale artificial bank filtration system. Therefore, this laboratory experiment provides a reference to the situation applied in the outdoor columns. Cerium concentrations in the outflow of the column were in the range of the limit of quantification, thus the transport of HA-CeO_2_ NP below 20 cm was very low. This demonstrates that a coating with HA was less stabilizing against aggregation and sedimentation than NOM indicating that different natural organic coatings vary in their stabilizing properties. This has also been shown by Li et al. (2017) who compared the stabilization of CeO_2_ NP by NOM of the Ohio River (OR) and Suwannee River-HA and found a stronger stabilization by OR-NOM. A recovery of 96 ± 8 % of Ce in the sediment proved the low transport of HA-CeO_2_ NP under soft surface water conditions (Online Resources, Table S1). The main part of the NP was retained in the laboratory columns already between 2 and 5 cm depth (Fig. 2b). The enhanced ionic strength in soft surface water compared to DI water caused a slight aggregation of HA-CeO_2_ NP (185 nm at the end of the experiment, Table 1). However, this particle size is too low to explain NP retention by mechanical filtration or straining processes (Bradford et al. 2002). It is more likely that chemical attachment processes, such as Ca^2+^ bridging or attachment in a deeper energy minimum were responsible for NP retention. As particles are mainly retained close to the column inlet the reversibility of attachment seems lower compared to NOM-CeO_2_ NP. However, highest Ce contents were not detected in the very first section of the column which suggests that HA-CeO_2_ NP are transported through the column very slowly following attachment and detachment processes. Such a bell-shaped retention profile has also been observed by Tong et al. (2005) for bacteria transport in quartz sand. The authors attributed this to detachment processes which are more pronounced at the column inlet due to the collision of attached and mobile colloids.

During our experiments, the colloidal stability of HA-CeO_2_ NP was confirmed by DLS measurements for a time period of 10 h. However, as shown by the column experiment, this cannot be directly related with a high potential for NP transport in sediment columns. Despite their high colloidal stability, HA-CeO_2_ NP transport was very low. An important reason for this is the presence of the sediment surfaces offering a high number of interaction sites for NP. Those SWI can lead to non-equilibrium attachment and therefore retention of NP as observed by Kumahor et al. (2016).

#### Hard surface water

Breakthrough of NOM-CeO_2_ NP was completely prevented in water with a Ca^2+^ concentration of 4.5 mM (Fig. 1c). Under these hydrochemical conditions, increased aggregate sizes were observed (Online Resources, Fig. S6). Potentially, this enhanced aggregation caused by NP destabilization led to physical straining processes, an additional retention mechanism besides adsorption to sediment surfaces. This can explain the accumulation of Ce in the upper 2 cm of the column (Fig. 2c). As demonstrated by Bradford et al. (2006), increasing ionic strength promotes straining and this process is most relevant close to the column inlet where particle aggregates enter small or dead pores and get retained. At a greater distance from the inlet, advection, dispersion, and size exclusion lead to flow focusing and therefore minimization of straining processes so that attachment is the determining retention process in deeper sediment layers.

Contrarily, PAA-CeO_2_ NP exhibited a total breakthrough of 58% under hard surface water conditions showing almost no retardation, which is comparable to the results in DI and soft surface water (Online Resources, Table S1). As for soft surface water conditions, a trend for increasing breakthrough over time is visible suggesting the saturation of attachment sites. The retention profile is also comparable to the one observed in soft surface water (compare Fig. 2b, c). These results prove the strong stabilizing effect of the synthetic coating agent.

For the transport experiment of NOM-CeO_2_ NP in hard surface water, we observed a very low recovery in sediment and water of only 18% of initially injected Ce. This might be attributed to the strong aggregation of the NP under high Ca^2+^ concentrations, which was observed immediately after mixing NP with the water (Online Resources, Fig. S6). Although the suspension was stirred over the whole experimental duration, not all NP may have reached the column due to sedimentation or aggregation in the tubes. Another possibility is that large CeO_2_ NP aggregates were caught in the glass frit (pore size 50 μm) at the bottom of the column. This would lead to a much lower input concentration of CeO_2_ NP than in the other experiments which might have reduced NP breakthrough. However, the complete amount of NP was immobilized at the beginning of the column due to the low colloidal stability of NOM-CeO_2_ NP. Therefore, we expect that even larger concentrations of CeO_2_ NP added to the column would have been retained in the very first part of the column.

Generally, our experiments showed a lower stabilization of CeO_2_ NP with natural organic coatings that consist of many different molecules leading to variable functional groups on NP surfaces, compared to PAA forming a layer of similar coating molecules on NP surfaces. A study by Geitner et al. (2017) showed contrary results with stronger stabilization provided by HA and NOM than by PVP. This was explained by the better steric stabilization by large, heterogeneous macromolecules than by uniform, linear molecules. However, PAA may have a very strong electrostatic stabilization potential that effectively stabilizes CeO_2_ NP as shown by Sehgal et al. (2005). Our results have important implications for the fate of NP in the environment. As industrial coatings are replaced or covered by natural organic molecules, their mobility might change dramatically (Louie et al. 2016). According to our results, a lower stability and hence, mobility, can be expected when natural coatings replace industrial ones, but this will depend on characteristics of both the industrial coating and the NOM present in the environment.

### Comparison of laboratory experiments with experiments in a semi-technical scale

In the outdoor sediment columns (SIMULAF), the insights from laboratory column experiments were compared with a system of conditions closer to a real-world scenario to value the relevance of data gained in small-scale and well-defined laboratory experiments. Besides the larger size of the experiment at semi-technical scale, the presence of natural colloids in the mobile phase and biological activity (e.g., presence of algae and biofilms) in the outdoor system represent major differences between lab and outdoor experiments.

**Fig. 3:**
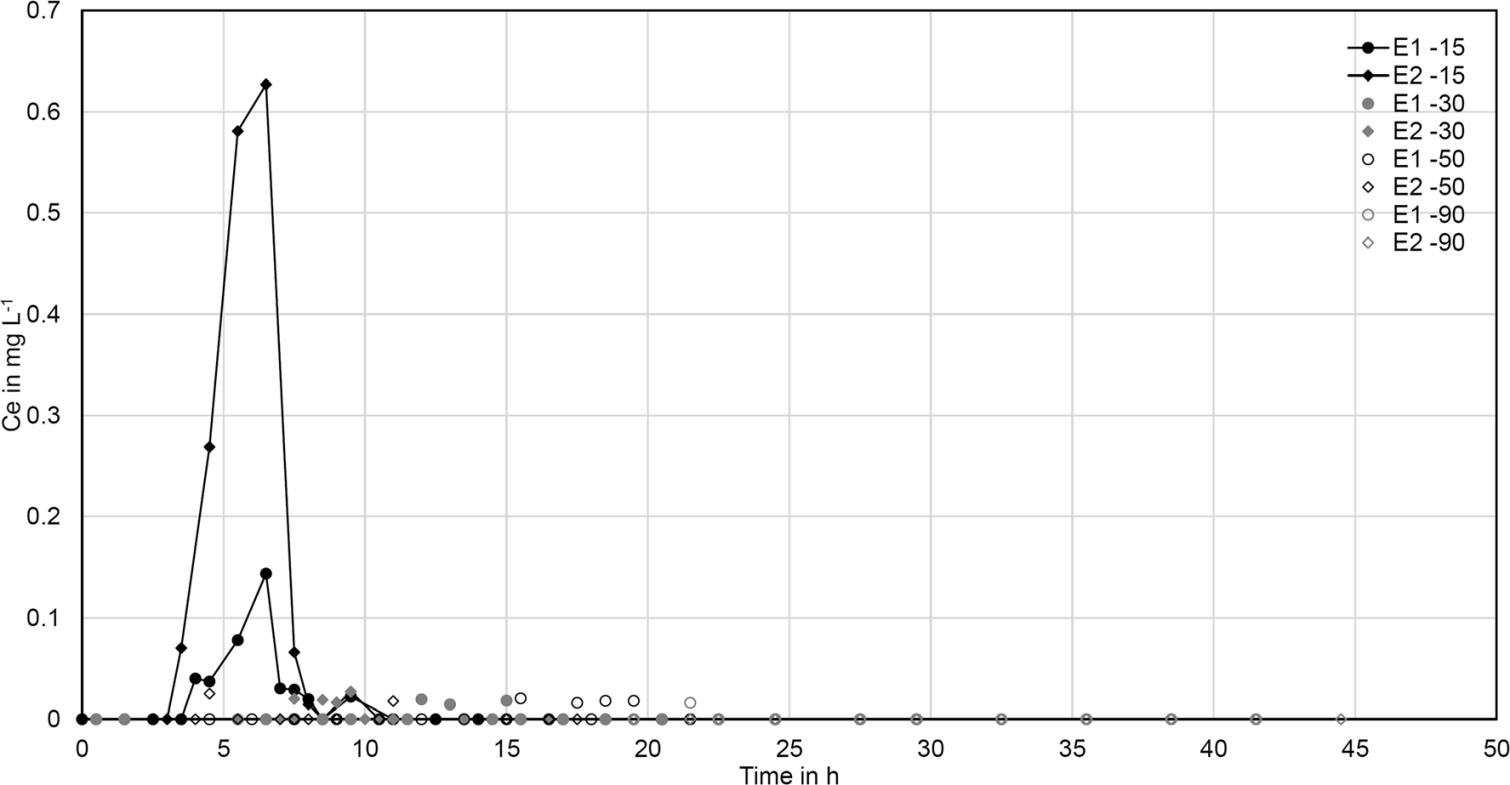
Cerium concentration in the pore water of two replicate columns (E1 and E2) at various sampling depths (15, 30, 50 and 90 cm) of outdoor experiments with HA-CeO_2_ NP. Breakthrough is illustrated over time and not pore volumes as pore volumes differ between the sampling depths while this graph demonstrates the time-shifted breakthrough in different depths

The injection of HA-CeO_2_ NP to the laboratory column led to a very low breakthrough suggesting a low risk for HA-CeO_2_ NP transport through sediment systems (Fig. 1b). This observation was confirmed in the outdoor bank filtration columns: only low amounts of Ce were quantified in the aqueous phase until a depth of 15 cm; in 30 and 50 cm depth, most concentrations were below the quantification limit, and in a depth of 90 cm, no Ce was detected except for one sampling point (Fig. 3). Thus, the main part of added HA-CeO_2_ NP was retained in the upper 15 cm.

**Fig. 4:**
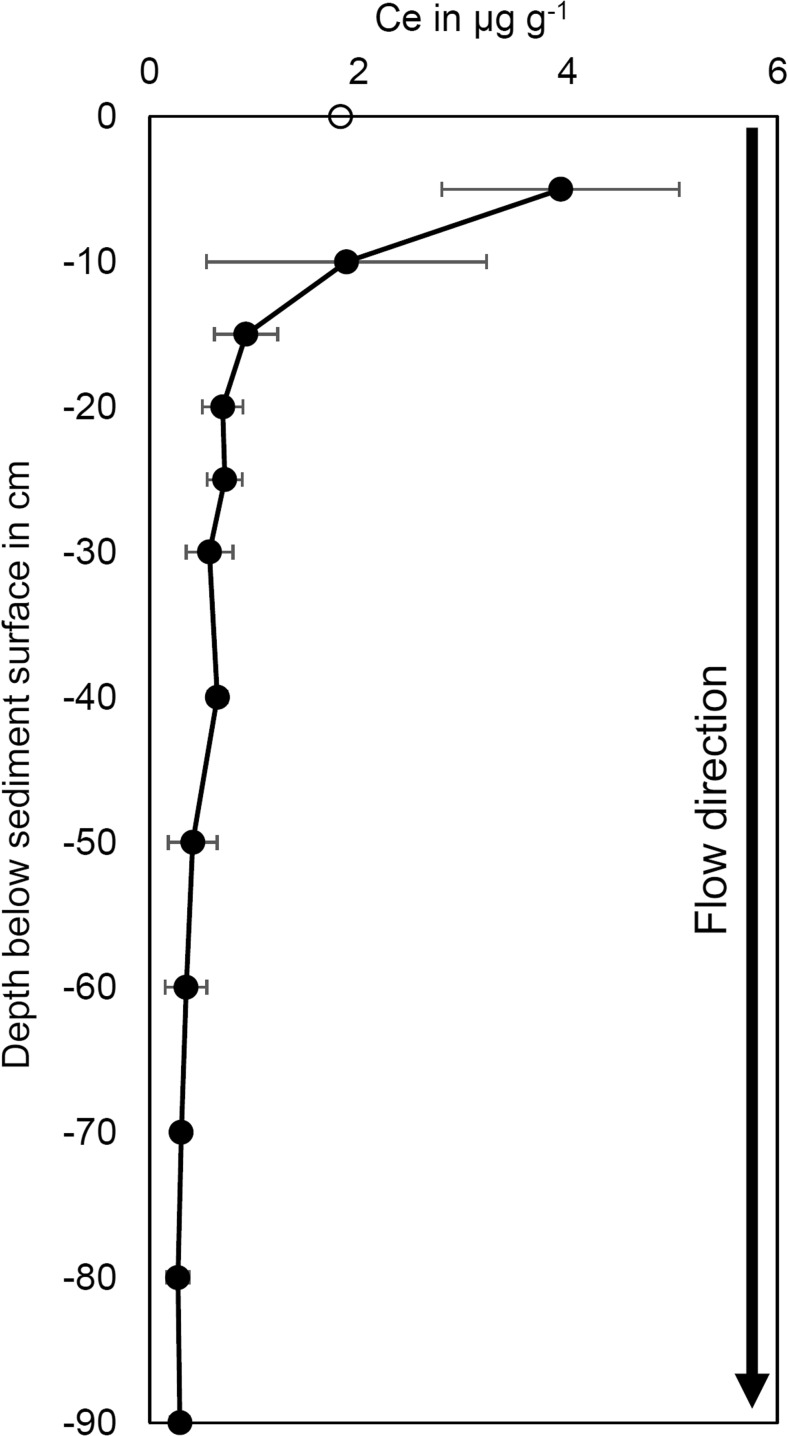
Cerium content in filter sand of the outdoor columns flushed with soft surface water for HA-CeO_2_ NP. Errorbars depict the range (maximum and minimum value) of 4 replicates (depth 0-30 cm) and 2 replicates (depth 40-90 cm) per column, respectively

In the sediments, highest Ce contents were found between a depth of 0 and 5 cm (Fig. 4), which is comparable to our findings in the laboratory (i.e., highest Ce contents between 2 and 5 cm depth, Fig. 2b). Even after an exchange of more than 400 PV in the upper 15 cm, most Ce was located in the top sediment layers which indicates a very low mobility and an irreversible attachment of HA-CeO_2_ NP on sediments of the artificial bank filtration system. In contrast to the lab columns, the calculation of a mass balance in the outdoor columns resulted in unrealistically high recoveries of 200–300% (Online Resources, Table S2). Due to the larger complexity of the outdoor system, CeO_2_ NP might have been more heterogeneously distributed in the sediment so that sampling was not representative. Additionally, the upper centimeters of the outdoor columns which retained the main part of the NP contained layers of organic deposits. They can reduce the bulk density of the upper sediment layers leading to an overestimation of total amounts of particles (Online Resources, Eq. 1). Detailed explanations can be found in the Online Resources. Overall, the accumulation and strong retention of CeO_2_ NP in the upper sediment layers was clearly visible from our experiment and confirmed lab-scale findings.

The accumulation of HA-CeO_2_ NP in the top sediment layers of outdoor columns was most likely attributed to the attachment of NP to solid surfaces. Straining is unlikely to occur as the NP were shown to be stable under the used hydrochemical conditions and therefore colloid sizes were too small to cause straining (Table 1). Instead, the interactions of NP with sediment surfaces (Kumahor et al. 2016) as well as with biofilms and plants (as shown by Ikuma et al. 2015; Tripathi et al. 2017) were probably important processes in the present experiment. Additionally, the presence of natural colloids is expected to play a central role in NP immobilization (Degenkolb et al. 2018; Hoppe et al. 2015). With a large surface area, colloids offer important attachment sites for NP in soils and are expected to reduce NP transport. However, the NP transport and retention behavior was comparable in both lab (i.e., absence of natural colloids) and outdoor (i.e., presence of natural colloids) experiments considering that the spatial resolution of sampling in the outdoor experiment was lower than in lab experiments. Hence, natural colloids were not determining for CeO_2_ NP retention in our study, although they may have interacted with the NP.

The Ce concentrations in the water of the bank filtration system at a depth of 15 cm differed considerably between the two sediment columns, which underlines the challenge of replicating tests in heterogeneous natural systems. Still, the trend in both columns was similar with a Ce breakthrough between 3.5 and 10 h in 15 cm depth, and no breakthrough below.

Changing weather conditions during the 3 weeks of the experiment did not influence NP breakthrough. Even a heavy rain event between days 6 and 8, which reduced the ionic strength in the water did not lead to NP remobilization and enhanced breakthrough (Online Resources, Fig. S4). In natural systems, heavy rain events may additionally cause enhanced flow velocities depending on the geological setting. As shown by Makselon et al. (2018), this can remobilize NP, but this factor was not included in our experiments as the pump rate was kept constant and saturated conditions were maintained at all times.

## Conclusions

With the laboratory column experiments, we could clearly observe differences between the transport of synthetically, PAA-coated CeO_2_ NP and natural organic matter-coated CeO_2_ NP: Changes in solution chemistry did not influence the breakthrough of the NP in the former case where generally a high breakthrough was observed. Instead, NP retention was strongly dependent on the water chemistry in the latter case where higher ionic strength and especially the presence of Ca^2+^ reduced the breakthrough of NOM-CeO_2_ NP and led to an accumulation of Ce already in the first centimeters of the column. We suggest that for both particles, the main retention process was chemical and electrostatic interaction with the solid phase instead of physical retention, such as straining or mechanical filtration. However, the NOM coating caused a strong sensitivity of CeO_2_ NP to hydrochemical changes leading to enhanced retention at higher ionic strength and Ca^2+^ concentration. In contrast, the PAA coating was less susceptible to increasing ionic strength and added colloidal stability to the NP and, thus, enhanced transport in water-saturated sediment systems. This is in line with our hypothesis that synthetically coated NP will be more mobile than naturally coated particles. However, the stabilizing manufactured coating might decompose or be exchanged against NOM in natural systems (Lau et al. 2013). Most likely, this would lead to a reduced transport in the environment.

The comparison of NOM-CeO_2_ NP with HA-CeO_2_ NP shows that different natural coating agents have different effects on the colloidal stability and transport of NP. Additionally, an important transport mechanism of PAA-coated CeO_2_ NP seems to be attributed to the high concentrations of excess PAA in suspension, which is not attached to NP surfaces. Entering the environment, a strong dilution of this background DOC is expected which would lead to lower NP transport than observed in our study. Therefore, future studies should exclude the effect of excess PAA by removing non-bound molecules before addition to the column.

Breakthrough of HA-CeO_2_ NP proved to be very low in both small-scale laboratory as well as large-scale near-natural experiments. In our experimental setup, changing weather conditions such as intense rain events were not the driving factor for NP transport. Furthermore, the presence of biological activity and natural colloids did not significantly change the transport behavior of HA-CeO_2_ NP. These results contradict our expectation of a stronger NP retention in more complex systems. Instead, we can conclude that important transport mechanisms for organically coated metal-oxide NP can already be described with less work-intensive but simplified laboratory experiments. In future studies, this should also be tested for a higher number of NP (e.g., PAA-CeO_2_ NP).

From the results of our study, we can conclude that an accumulation of naturally coated CeO_2_ NP in surficial sediment layers of bank filtration systems can be expected. These upper sediment layers might be exposed to shear forces, water turbulences, and changing hydrochemical conditions when in contact with the river bed. Possibly, this might lead to remobilization of CeO_2_ NP when physicochemical conditions change, a mechanism which was already demonstrated for Ag NP by Degenkolb et al. (2018).

## Electronic supplementary material

ESM 1 (PDF 972 kb)
