# Supplementary material for: Transport and retention of differently coated CeO2 nanoparticles in saturated sediment columns under laboratory and near-natural conditions
Source: Environ Sci Pollut Res Int. 2019 Apr 8;26(16):15905–19. doi: 10.1007/s11356-019-04965-x (PMC6533415; doi:10.1007/s11356-019-04965-x)
Supplement: Supplementary file 1 — (PDF 972 kb) [file 11356_2019_4965_MOESM1_ESM.pdf]

Online Resources

Article: Transport and retention of differently coated CeO_2 nanoparticles in saturated sediment columns under laboratory and near-natural conditions

Journal: Environmental Science and Pollution Research

Authors:
Laura Degenkolb[a,b,$]
laura.degenkolb@uba.de
Urs Dippon[a,$]
urs.dippon@uba.de
Silke Pabst[a]
silke.pabst@uba.de
Sondra Klitzke[a]
sondra.klitzke@uba.de

a) German Environment Agency, Section Drinking Water Treatment and Resource Protection, Schichauweg 58, 12307 Berlin, Germany
b) Berlin University of Technology, Institute of Ecology, Department of Soil Science, Ernst-Reuter Platz 1, 10587 Berlin, Germany
$) Both authors contributed equally.

Corresponding author: Urs Dippon: urs.dippon@uba.de

List of Figures

Fig. S 1 Concentration of the main ions in the hard surface water over time of the experiments. This water was used for both lab and outdoor experiments either undiluted or diluted using DI water ... 3

Fig. S 2 Grain size distribution of the filtersand used in column experiments ... 4

Fig. S 3 Schematic drawing of the outdoor column at the SIMULAF facility including positions of the sampling ports (P0 - P4) ... 4

Fig. S 4 Precipitation, electric conductivity and water temperature during the bank filtration experiment. Section marked with “t” illustrates the time difference in the drop of electric conductivity between the inflow and the outflow of the artificial bank filtration system ... 5

Fig. S 5 Methods for the extraction of cerium from filter sand. Left panel: full extraction of 1 g filter sand by reverse aqua regia (3:1 HNO_3 / HCl) digestion without and with spiking of 1 mg cerium in form of CeO_2 NP. The cerium background is 3 to 16 times higher than cerium concentration added as CeO_2 NP. Right panel: Selective extraction of cerium from added CeO_2 NP (1 mg L^{-1}) by physical and chemical methods. Best recovery and lowest cerium background were gained by ultrasonic bath treatment for 60 min (red frame). Grey shaded concertation range: quantification limit. NH_4-Ac = ammonium acetate ... 5

Fig. S 6 Time-dependent aggregation of NOM- CeO_2 NP in hard surface water, error bars depict standard deviations of 3-6 replicates ... 7

List of tables

Table S 1: Total recovery and breakthrough of differently coated CeO_2 NP in the laboratory column experiments in different types of water ... 3

Table S 2: Mass balance of outdoor columns, recovery of Ce in the sediment of the sand filtration columns ... 7

Table S 1: Total recovery and breakthrough of differently coated CeO_2 NP in the laboratory column experiments in different types of water

**Medium**	**DI water**		**Soft surface water**			**Hard surface water**	
**Coating**	**PAA**	**NOM**	**PAA**	**NOM**	**HA**	**PAA**	**NOM**
**Recovery (mean) [%]**	107.44	92.68	71.33	75.83	96.27	97.01	18.17
**SD**	4.40	4.24	3.47	1.58	7.64	11.68	1.11
**Breakthrough (mean) [%]**	60.27	27.23	50.70	6.05	1.30	58.32	0.00
**SD**	1.13	0.74	4.35	0.15	0.00	6.27	0.00

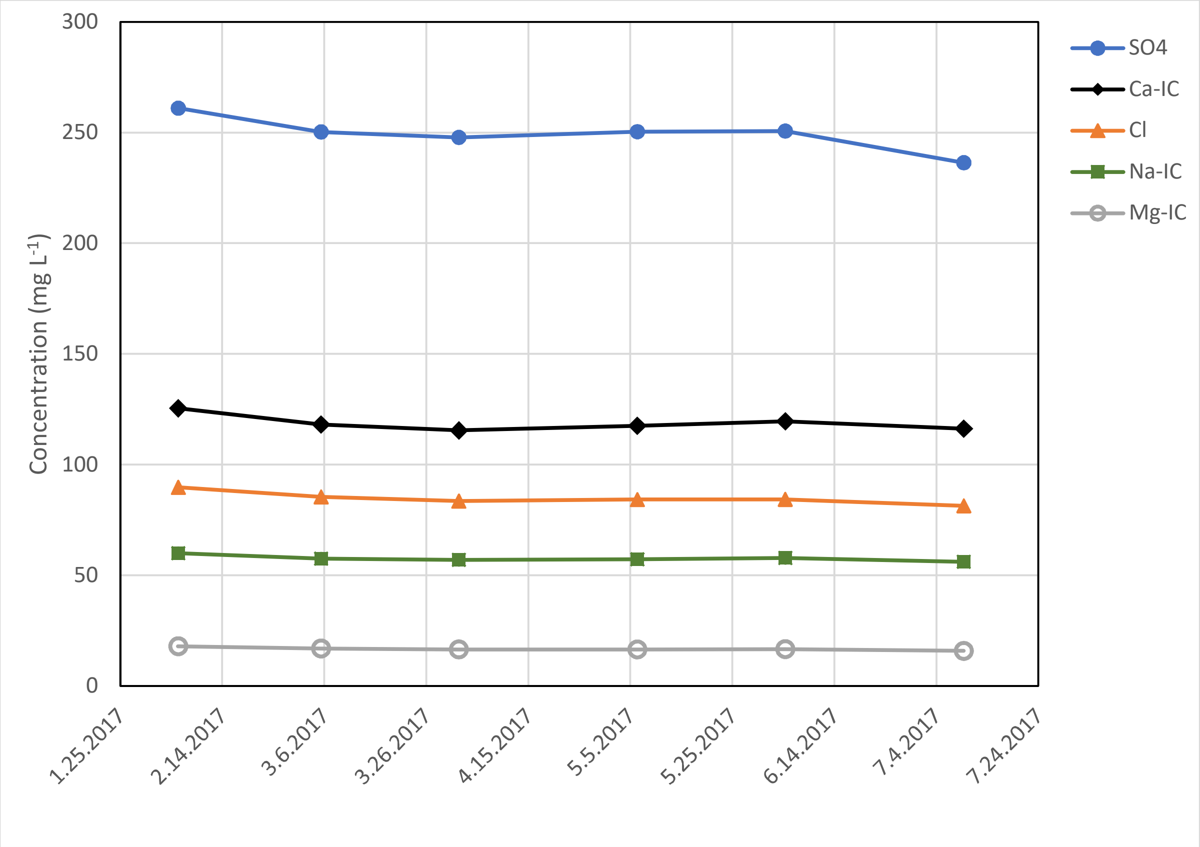

Fig. S 1 Concentration of the main ions in the hard surface water over time of the experiments. This water was used for both lab and outdoor experiments either undiluted or diluted using DI water

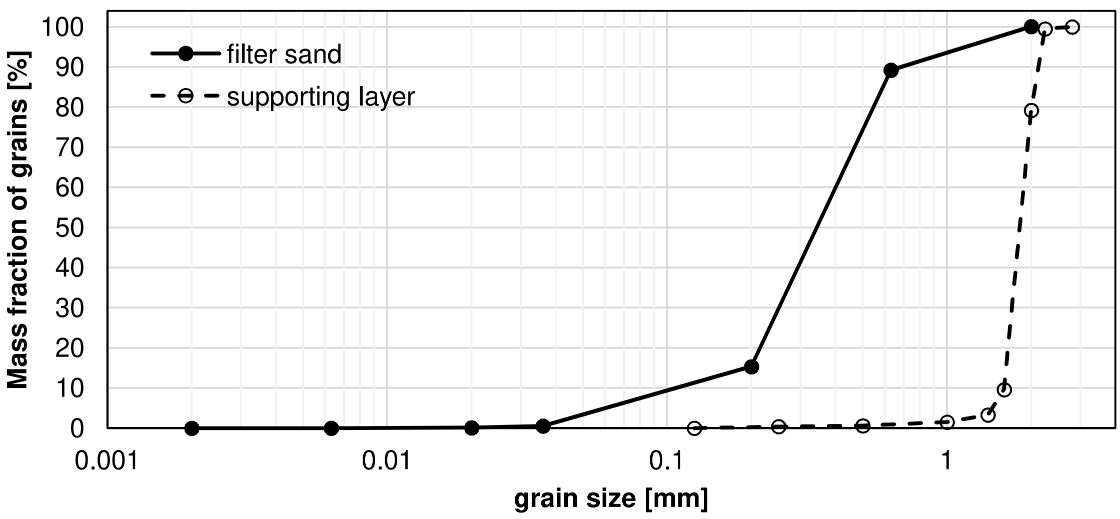

Fig. S 2 Grain size distribution of the filtersand used in column experiments

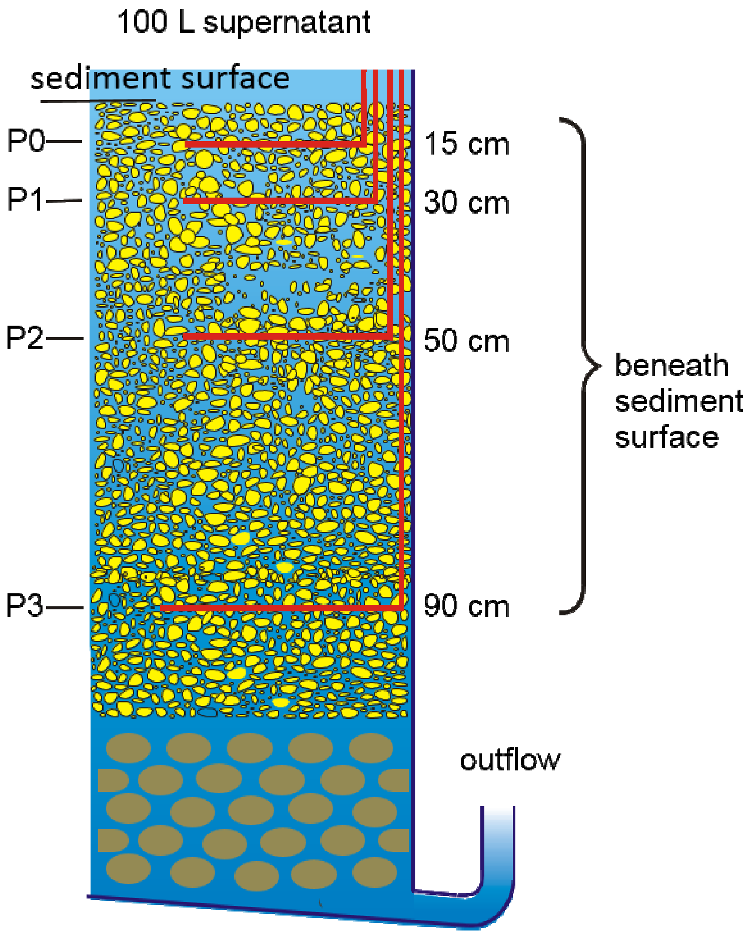

Fig. S 3 Schematic drawing of the outdoor column at the SIMULAF facility including positions of the sampling ports (P0 - P4)

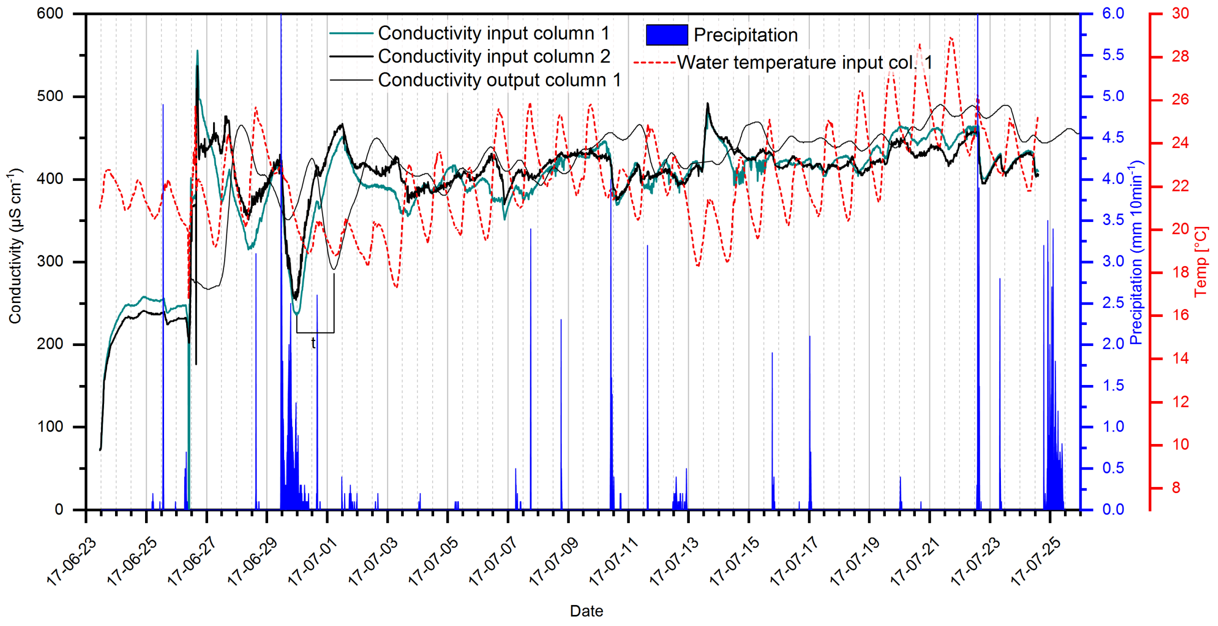

Fig. S 4 Precipitation, electric conductivity and water temperature during the bank filtration experiment. Section marked with "t" illustrates the time difference in the drop of electric conductivity between the inflow and the outflow of the artificial bank filtration system

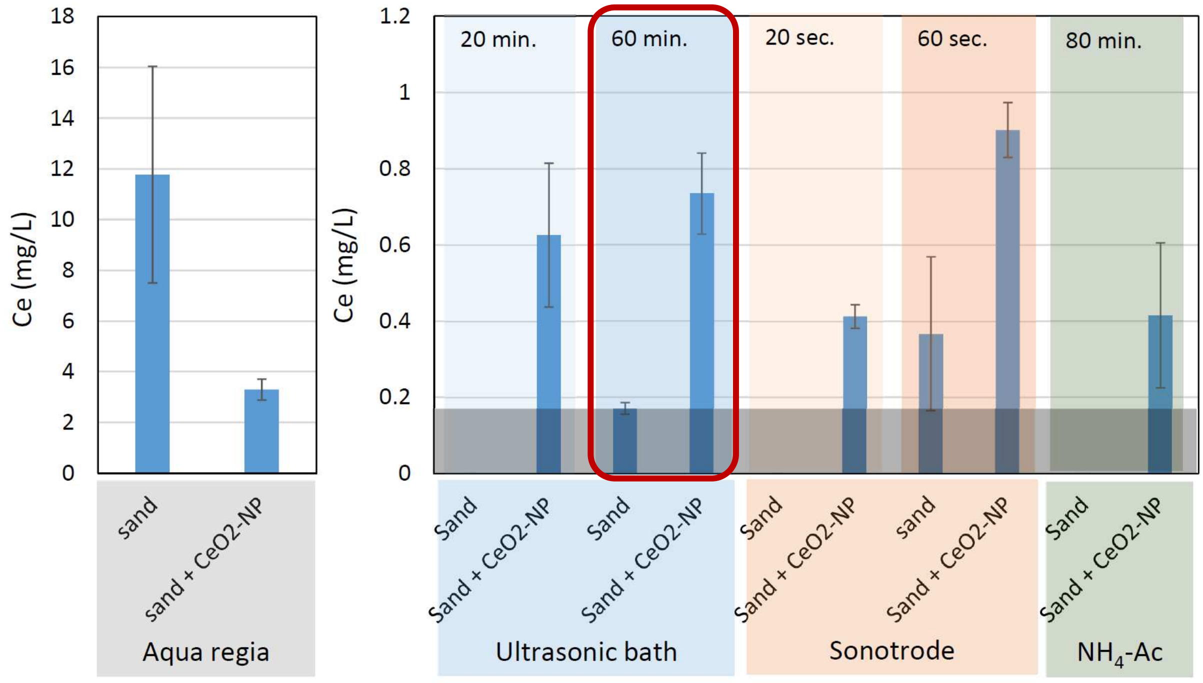

Fig. S 5 Methods for the extraction of cerium from filter sand. Left panel: full extraction of 1 g filter sand by reverse aqua regia (3:1 HNO_3 / HCl) digestion without and with spiking of 1 mg cerium in form of CeO_2 NP. The cerium background is 3 to 16 times higher than cerium concentration added as CeO_2 NP. Right panel: Selective extraction of cerium from added CeO_2 NP (1 mg L^{-1}) by physical and chemical methods. Best recovery and lowest cerium background were gained by ultrasonic bath treatment for 60 min (red frame). Grey shaded concertation range: quantification limit. NH_4-Ac = ammonium acetate

Calculation of mass balance: The total recovery of CeO_2 NP in all experiments was calculated from the Ce mass broken through with the mobile phase and the Ce content in the sediments (Eq. 1). For the latter one, a recovery of 80 % during the digestion method was assumed.

$$R_{total} = \frac{a*\sum_{n=1}^{t}(c_n*V_n)+b*\sum_{m=1}^{s}(c_m*V_m)}{m_{Ce}} \qquad \text{Eq. 1}$$

t	sediment sampling depth
s	number of water sample
c_n	concentration of Ce in sediment sample n
V_n	sediment volume in sampling depth n
c_m	concentration of Ce in effluent sample m
V_m	water volume of sample m
m_{Ce}	mass of Cerium applied to the respective column experiment
a	correction factor for porosity (0.35), material density (2.65 g cm^{-3}), and digestion recovery (80%)
b	correction factor for sampling volume (10 mL) and total volume (i.e. flow through)

The total amounts of Ce found in the column of outdoor experiments exceed the amount of Ce added to the column. Therefore, some assumptions of the calculation of the mass balance might not be appropriate for our outdoor column experiment. First of all, we analyzed only a small part of the sediments and found large differences between different sampling points. Therefore, it is possible that we took samples in areas with higher Ce accumulation than in other regions of the sampling. Recalculating the measured Ce contents in sediments to the total amount of Ce in the columns we assumed that this concentration can be found in the complete sediment layer, which then led to extremely high Ce concentrations exceeding the total amount applied to the columns. Therefore, we suggest that unrepresentative sampling can be one reason for the high mass balance. The second factor that might have led to an overestimation of total Ce in the column is the presence of C in the upper sediment layer. This reduced the bulk density, which was not corrected for in the calculation of our mass balance. As we multiplied the Ce content in µg g^{-1} with the volume of the sediment layer (Volume = bulk density*layer height*column surface area), an overestimated bulk density leads to overestimation of Ce content in the sediment. However, we tried to

recalculate with a corrected, smaller bulk density and found that this influence is only of minor importance and other factors must be involved.

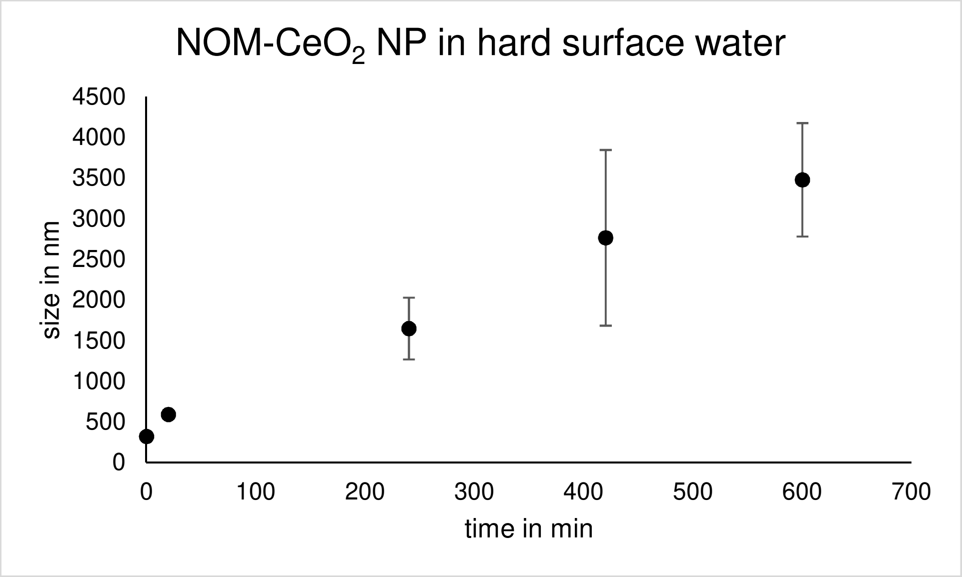

Fig. S 6 Time-dependent aggregation of NOM- CeO_2 NP in hard surface water, error bars depict standard deviations of 3-6 replicates

Table S 2: Mass balance of outdoor columns, recovery of Ce in the sediment of the sand filtration columns

**Column (Depth)**	**E1 (0-75 cm)**	**E2 (0-75 cm)**	**E1 (0-30 cm)**	**E2 (0-30 cm)**
**Recovery [%]**	283.95	240.22	155.05	236.46
